# Integrated Analysis Reveals the Gut Microbial Metabolite TMAO Promotes Inflammatory Hepatocellular Carcinoma by Upregulating POSTN

**DOI:** 10.3389/fcell.2022.840171

**Published:** 2022-05-23

**Authors:** Yonglin Wu, Xingyu Rong, Miaomiao Pan, Tongyao Wang, Hao Yang, Xiejiu Chen, Zhenming Xiao, Chao Zhao

**Affiliations:** ^1^ Key Laboratory of Medical Molecular Virology (MOE/NHC/CAMS), School of Basic Medical Sciences, Shanghai Medical College and National Clinical Research Center for Aging and Medicine, Shanghai Medical College, Huashan Hospital, Fudan University, Shanghai, China; ^2^ Shanghai Frontiers Science Center of Pathogenic Microbes and Infection, Shanghai, China

**Keywords:** microbial metabolite, TMAO, inflammatory, hepatocellular carcinoma, RNA-seq, database reanalysis

## Abstract

Liver cancer has a high mortality rate. Chronic inflammation is one of the leading causes of hepatocellular carcinoma. Recent studies suggested high levels of trimethylamine N-oxide (TMAO) may correlate with increased risk of inflammatory-induced liver cancer. However, the mechanisms by which TMAO promotes liver cancer remain elusive. Here, we established a model of inflammatory-induced liver cancer by treating Hepa1-6 cells and Huh7 cells with TNF-α. TMAO synergistically increased the proliferation, migration and invasion of Hepa1-6 cells and Huh7 cells in the presence of TNF-α. We conducted bulk RNA-Seq of the TMAO-treated cell model of inflammatory Hepatocellular carcinoma (HCC) and evaluated the influence of the differentially expressed genes (DEGs) on clinical prognosis using Kaplan-Meier Plotter Database and Gene Expression Profiling Interactive Analysis (GEPIA) database. Univariate and multivariate Cox regression analyses of tumor microenvironment and DEGs were performed using Timer2.0. Upregulation of *POSTN*, *LAYN* and *HTRA3* and downregulation of *AANAT* and *AFM* were positively related to poorer overall survival in human liver cancer. Moreover, higher expression of *POSTN* and *HTRA3* positively correlated with infiltration of neutrophils, which can promote tumor progression. *In vitro* experiments showed TMAO activates ILK/AKT/mTOR signaling via *POSTN*, and knocking down *POSTN* significantly reduced ILK/AKT/mTOR signaling and the tumorigenicity of Hepa1-6 cells and Huh7 cells. Collectively, our results suggest the gut microbial metabolite TMAO and *POSTN* may represent potential therapeutic targets for liver cancer.

## Introduction

Although liver cancer is the sixth most common cancer and has the third highest cancer mortality rate (Sung et al., 2021). Hepatocellular carcinoma (HCC) is the most common type of primary liver cancer (PLC), accounting for 70%–80% of cases (Siegel et al., 2018). The first-line treatment option for HCC is liver transplantation. Local ablation and external radiation are the standard of care for patients who are not suitable for surgery. Sorafenib and lenvatinib are the standard first-line systemic therapies for HCC, but are only indicated for patients with well-preserved liver function (EASL, 2018).

HCC is strongly associated with alcohol consumption, viral hepatitis such as hepatitis B virus (HBV) or hepatitis C virus (HCV), and nonalcoholic fatty liver disease (NAFLD) (Ghouri et al., 2017; Rong et al., 2019). All of these factors alter the hepatic immune system and induce severe chronic liver inflammation via multiple pathways and cytokine signaling molecules (Cui et al., 2017; Yang YM. et al., 2019). Tumor necrosis factor-alpha (TNF-α) is a pro-inflammatory Th1 cytokine that plays critical roles in the inflammatory mechanisms implicated in HCC (Jing et al., 2018). A recent study reported that TNF-α induces activation of the TNFR2-hnRNPK-YAP signaling axis and promotes primary liver tumorigenesis, confirming the important role of TNF-α in the pathogenesis of malignancies and inflammatory autoimmune diseases (Meng et al., 2021). Thus, TNF-α functions as a critical regulator of the induction of inflammatory HCC.

Trimethylamine N-oxide (TMAO) is an oxidation product of trimethylamine (TMA) and a pro-atherosclerotic metabolite. The role of TMAO in the relationship between metabolism of dietary compounds by the microbiota and cardiovascular disease has been investigated. When choline and other trimethylamine-containing species are ingested, TMA is initially formed by initial catabolism by intestinal microbes, and then efficiently metabolized by the hepatic flavin monooxygenase (FMO) family of enzymes to form TMAO (Wang et al., 2011). TMAO is also strongly implicated in development of the chronic inflammatory disease atherosclerosis (AS). Elevated TMAO levels induce activation of the NF-kappa B (NF-κB) pathway, which contributes to the regulation of many AS-related genes (Baker et al., 2011; Ma et al., 2017; Cheng et al., 2019). In addition, TMAO also increases the expression of pro-inflammatory genes, such as inflammatory factors, adhesion molecules and chemokines, in various models (Rohrmann et al., 2016; Chen ML. et al., 2017). TMAO can also activate the NLRP3 inflammasome and induce oxidative stress (Sun et al., 2016; Boini et al., 2017). Thus, TMAO undeniably contributes to the occurrence of chronic inflammation.

Numerous investigations have strongly linked TMAO to colorectal cancer and other malignant diseases (Bae et al., 2014; Chen et al., 2016; Shan et al., 2017). However, few studies have explored the association between TMAO and HCC. A recent case-control study indicated a relationship between TMAO and its precursor choline with HCC, with higher TMAO concentrations associated with increased risk of HCC (Liu et al., 2018). Here, used Hepa1-6 and Huh7 cells to establish the inflammatory hepatocellular carcinoma model promoted by TNF-α to explore the relationship between TMAO and the pathogenesis of inflammatory HCC promoted by TNF-α.

## Materials and Methods

### Cell Lines

The mouse HCC cell lines Hepa1-6 was purchased from American type culture collection (ATCC) and human HCC cell line Huh7 was purchased from JCRB cell bank which were both maintained in Dulbecco’s modified Eagle’s medium (DMEM) with 10% fetal bovine serum (FBS).

### TNF-α and TMAO

Recombinant mouse TNF-α and recombinant human TNF-α were purchased from PeproTech and dissolved in FBS. TMAO was purchased from Sigma-Aldrich and dissolved in DMEM with 10% FBS.

### Cell Viability Assay

Cell viability was evaluated using the Cell Counting Kit-8 (CCK-8; Dojindo, Shanghai, China) in accordance with the manufacturer’s instructions. Briefly, cells (2 × 10^3^) were seeded into 96-well plates and cultured for 24, 72 or 120 h. To generate a growth curve, 10 μL CCK-8 solution was added to each well, incubated at 37°C protected from light for 4 h, and optical density was measured at 450 nm.

### Wound Healing Assay

Cells (2 × 10^5^) were seeded into 12-well plates and cultured to produce confluent monolayers. Wound areas were scraped using 200 μL pipette tips, washed three times with PBS to remove cellular debris and then the cells were cultured in DMEM with 10% FBS. Wound closure was observed and photographed between 0 and 48 h under an inverted microscope.

### Migration Assays

The migration assay was performed using Transwell chambers (cat. no. 3422; Corning Incorporated, Shanghai, China). Briefly, 8 × 10^4^ cells were centrifuged, resuspended in 250 µL serum-free media and placed into the upper chamber, and 750 µL of media containing 10% FBS was added to the lower chamber. The plates were incubated at 37°C for 24 h, then the culture media in the upper chamber was discarded, and the cells in the lower chamber were fixed with 600 µL of 4% paraformaldehyde for 20 min, washed with PBS, and stained in 400 µL of 0.1% crystal violet for 10 min. The numbers of cells that had migrated through the polycarbonate membrane at 6 h and invaded at 48 h were observed in at least five randomly selected fields under an inverted light microscope.

### Transcriptome Sequencing

Total RNA was extracted, and mRNA was isolated using Oligo Magnetic Beads and fragmented for cDNA synthesis. RNA-seq cDNA libraries were prepared and sequenced on an Illumina sequencing platform in 2*150 sequencing mode.

### Gene Correlation Analysis in GEPIA

The online database Gene Expression Profiling Interactive Analysis (GEPIA) (http://gepia.cancer-pku.cn/index.html) is an interactive web resource that includes RNA sequencing and patient outcome data for 9,736 tumor and 8,587 normal samples from the TCGA and GTEx projects. GEPIA was used to generate overall survival (OS) based on gene expression levels in HCC; the curves were compared using the log-rank test and Mantel-Cox test. Gene expression correlation analysis was also performed for selected TCGA expression datasets, using the tumor and normal tissue datasets.

### Kaplan-Meier Plotter Database Analysis

Kaplan-Meier plotter can be used to assess the effects of 54,675 genes on patient survival in 10,461 cancer samples (http://kmplot.com/analysis/). The correlations between *POSTN*, *NAPB*, *LAYN*, *HTRA3*, *AFM* and *AANAT* expression and patient survival in liver cancer were analyzed with Kaplan-Meier plotter; using the best cut-off analysis for stratification of HCC patients; the hazard ratios (HR) with 95% confidence intervals and log-rank p-values were computed.

### TIMER 2.0 Database Analysis

TIMER 2.0 is a comprehensive resource for systematic analysis of immune infiltrates across diverse cancer types (https://cistrome.shinyapps.io/timer/). TIMER 2.0 applies deconvolution via a previously published statistical method to infer the abundance of tumor-infiltrating immune cells (TIICs) from gene expression profiles. The TIMER 2.0 database includes 10,897 samples across 32 cancer types from The Cancer Genome Atlas (TCGA) and can be used to estimate the abundance of immune infiltrates. We analyzed the correlations between the levels of infiltration of neutrophils, Tregs, activated NK cells, and CD8^+^ T cells and the expression levels of *POSTN, NAPB, LAYN, HTRA3, AANAT,* and *AFM* in liver cancer*.*

### Quantitative Real-Time PCR

Cellular and exosomal RNAs were isolated using Eastep® Super Total RNA Extraction Kit (Promega, Madison, Wisconsin United States). First-strand cDNA was synthesized with random primers using Eastep® RT Master Mix (Promega). QPCR was performed with GoTaq® qPCR Master Mix (Promega) on a CFX384 Real-Time PCR Detection System (Bio-Rad, Hercules, California, United States). The primers were synthesized by BGI Genomics (Shanghai, China) and are listed in Additional file: Supplementary Table S1. Relative quantification was performed with the 2^-∆∆Ct^ method.

### Western Blotting

Total cellular proteins or exosomes were extracted with RIPA buffer (Sigma-Aldrich, St. Louis, Missouri, United States) and protein concentrations were quantified using the BCA assay (Pierce, Rockford, IL, United States). Total proteins were separated on polyacrylamide gels, transferred onto PVDF membranes, blocked with TBS-T solution containing 5% BSA for 1 h, then the membranes were incubated with primary antibodies against POSTN (sc-398631, Santa Cruz; 1:500), NAPB (A18223, ABclonal; 1:1000), HTRA3 (NB600-1151, Novus Biologicals; 1:1000), LAYN (ab192610, Abcam; 1:1000), ILK (3862, Cell Signaling Technology; 1:1000), AKT (4691, Cell Signaling Technology; 1:1000), p-AKT (4060, Cell Signaling Technology; 1:1000), mTOR (2983, Cell Signaling Technology; 1:1000) and p-mTOR (5536, Cell Signaling Technology; 1:1000). Membranes were incubated with secondary antibodies including Anti-rabbit IgG (7074, Cell Signaling Technology; 1:10000) and Goat Anti-Mouse IgG H&L (ab6789, Abcam; 1:10000) for 1 h at room temperature. The western blots were visualized using an enhanced chemiluminescence system.

### Production of Postn-Knockdown Recombinant Lentivirus Particles

The sh-*postn*-containing transfer vectors sh-*postn*-1, sh-*postn*-2 and sh-*postn*-3 were purchased from Shanghai Genechem Co., LTD. and co-transfected into 293T cells using Lipofectamine^TM^ 3000, together with the pMD2G and psPAX2 plasmids. Forty-eight hours later, the supernatant was collected and replaced with fresh media, and the supernatant was collected again 72 h later. The supernatants containing the lentivirus particles were gently mixed, centrifuged at 4,000 g for 5 min at 4°C, and stored at −70°C for use in subsequent experiments.

### Knockdown of Postn in Hepa1-6 Cells and Huh7 Cells

Hepa1-6 cells (2 × 10^5^) and Huh7 cells were seeded into 12-well plates and cultured to 70%–90% confluence, then the virus supernatant containing the lentivirus particles was added and the cells were incubated for 24 h Hepa1-6 cells and Huh7 cells infected with the *Postn*-knockdown lentivirus were selected by culture in puromycin for 2 weeks.

### Statistical Analysis

The Student’s *t*-test was used to compare groups for normally distributed data; otherwise, the nonparametric Mann-Whitney test was adopted. One-way ANOVA was applied to compare the differences among three groups. For survival analysis, univariate analysis was conducted by the Kaplan-Meier method with the log-rank test, and multivariate analysis was performed by the stepwise Cox multivariate proportional hazard regression model (Forward LR, likelihood ratio). All *in vitro* experiments were replicated three times. Statistical analyses were performed using Graphpad prism 8.0 software, all the tests are two-sided; *p* values <0.05 were considered statistically significant.

## Results

### TMAO Synergistically Enhances the Tumorigenicity of Hepa1-6 Cells in the Presence of TNF-α

**FIGURE 1:**
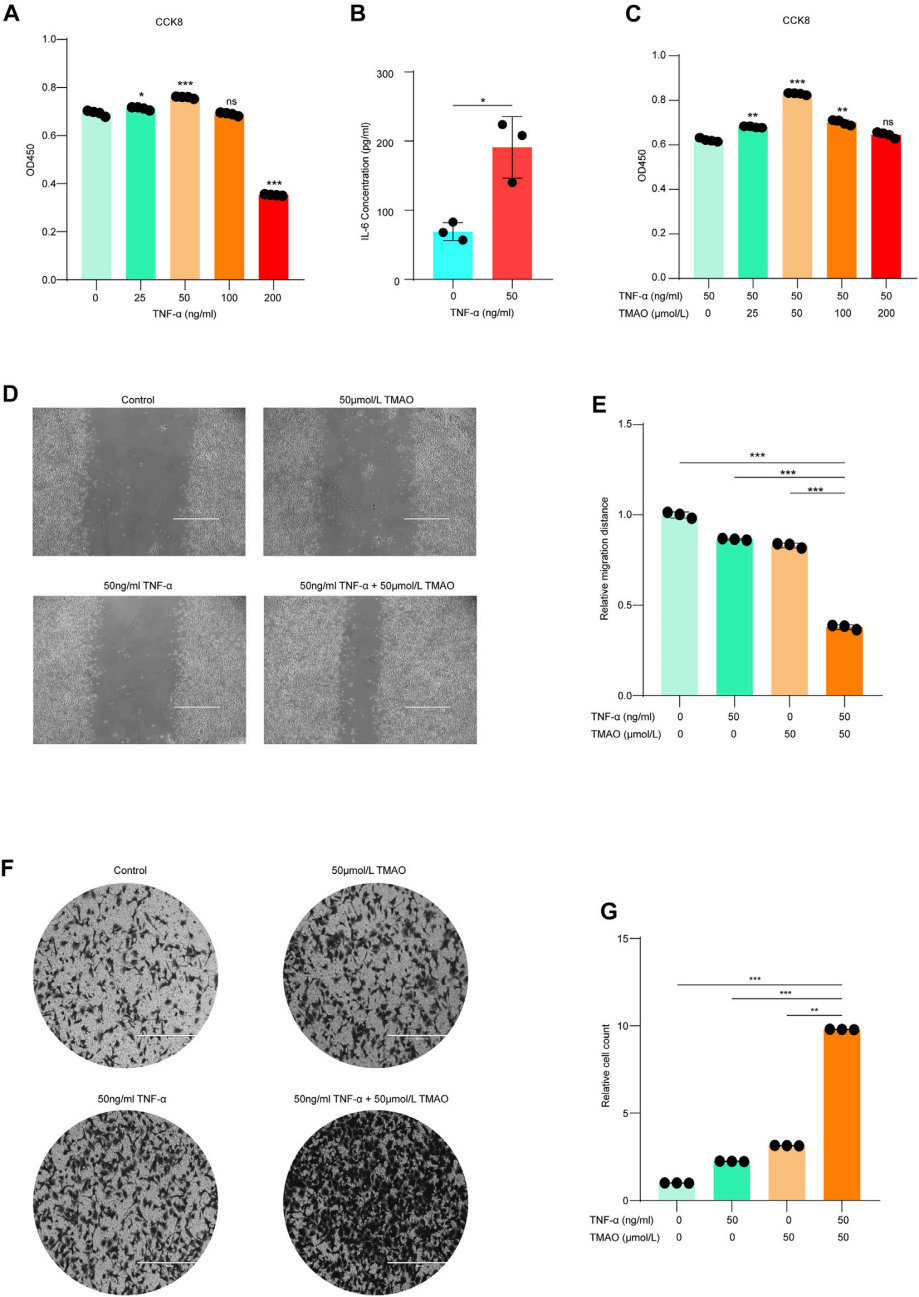
TMAO synergistically enhances the tumorigenicity of Hepa1-6 cells in the presence of TNF-α. **(A)** TNF-α dose-dependently promoted the proliferation of Hepa1-6 cells; maximum proliferation was observed at 50 ng/ml TNF-α; results are mean ± SD (*n* = 3 per group); one-way ANOVA with Bonferroni’s test; ***, *p* < 0.001. **(B)** Hepa1-6 cells treated with 50 ng/ml TNF-α expressed higher levels of IL-6 compared to untreated cells; results are mean ± SD (*n* = 3 per group); Student’s t-test; ***, *p* < 0.001. **(C)** The combination of 50 ng/ml TNF-α and 50 μM TMAO led to the maximal synergistic increase in cell proliferation; results are mean ± SD (*n* = 3 per group); one-way ANOVA with Bonferroni’s test; ***, *p* < 0.001. **(D,E)** Wound healing assay confirmed that 50 ng/ml TNF-α alone and 50 μM TMAO alone slightly promoted the migration of Hepa1-6 cells compared to untreated control cells, while the combination of 50 ng/ml TNF-α and 50 μM TMAO synergistically and significantly promoted the migration of Hepa1-6 cells. Scale bars = 1,000 μm; data were analyzed by ImageJ; data are mean ± SD (*n* = 3 per group); one-way ANOVA with Bonferroni’s test. **(F, G)** Migration assay confirmed that 50 ng/ml TNF-α alone and 50 μM TMAO alone slightly promoted the migration of Hepa1-6 cells compared to untreated control cells, while the combination of 50 ng/ml TNF-α and 50 μM TMAO synergistically and significantly promoted the migration of Hepa1-6 cells. Scale bars = 400 μm; data were analyzed by ImageJ; data are mean ± SD (*n* = 3 per group); one-way ANOVA with Bonferroni’s test.

TNF-α is a proinflammatory cytokine that has been linked to the initiation and development of HCC. Indeed, we found that TNF-α dose-dependently promoted the proliferation of Hepa1-6 mouse hepatoma cells, with the highest proliferation observed at 50 ng/ml TNF-α (Figure 1A). To investigate whether TNF-α promotes Hepa1-6 cell proliferation in an inflammatory state, we measured the levels of the inflammatory hepatocellular carcinoma-related cytokine IL-6 in Hepa1-6 cells treated with 50 ng/ml TNF-α. The levels of IL-6 were higher in Hepa1-6 cells treated with 50 ng/ml TNF-α compared to untreated cells (Figure 1B).

Interestingly, we also found that 50 ng/ml TNF-α synergistically promoted the proliferation of Hepa1-6 cells in combination with specific concentrations of trimethylamine N-oxide (TMAO), a co-metabolite of the liver and gut microbiota (Figure 1C). After screening the optimal doses of TNF-α and TMAO, four treatment groups were established to further investigate the effects of TNF-α and TMAO on the function of hepatoma Hepa1-6 cells: no treatment control, 50 ng/ml TNF-α alone, 50 μM TMAO alone, and the combination of 50 ng/ml TNF-α and 50 μM TMAO.

Wound healing and migration assays confirmed that either 50 ng/ml TNF-α or 50 μM TMAO alone slightly promoted the migration of Hepa1-6 cells compared with control cells; however, the combination of 50 ng/ml TNF-α and 50 μM TMAO synergistically and significantly promoted the migration of Hepa1-6 cells (Figures 1D–G).

### Transcriptomic Alterations in TMAO-Induced Primary Liver Cancer Cell Lines

**FIGURE 2:**
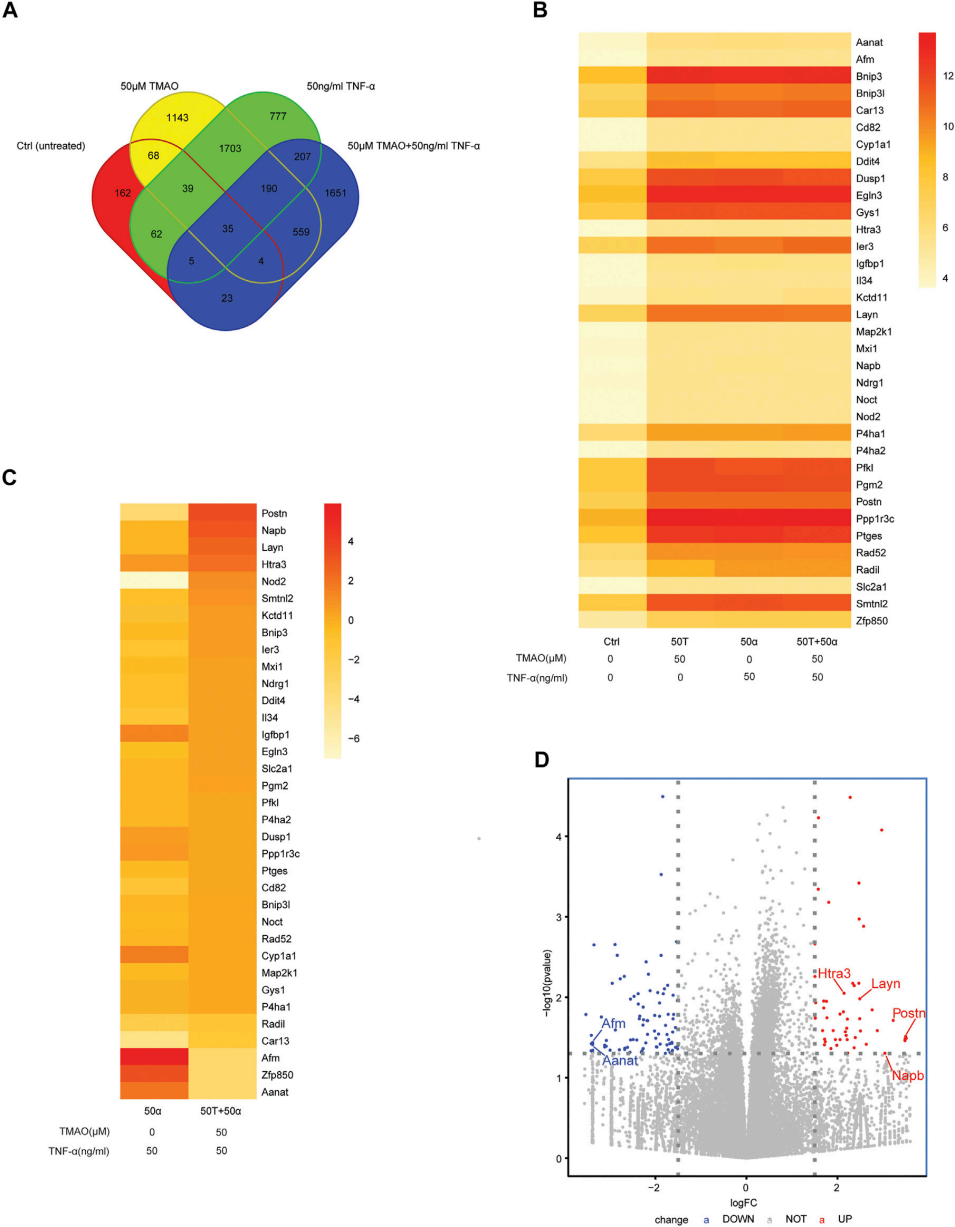
Transcriptomic alterations in TMAO-induced primary liver cancer cells. **(A)** Venn diagrams showing the crossover between the differentially expressed genes (DEGs) in untreated Hepa1-6 cells (Group Ctrl), and Hepa1-6 cells treated with 50 μM TMAO alone (Group 50T), 50 ng/ml TNF-α alone (Group 50α) or 50 ng/ml TNF-α and 50 μM TMAO in combination (Group 50T + 50α). **(B)** Heatmap showing the DEGs common to the four treatment groups; FC > 2 and *p <* 0.05. **(C)** Heatmap showing the DEGs common to Group 50α and Group 50T + 50α; FC > 2 and *p <* 0.05. **(D)** Volcano map showing the significant differentially expressed genes between Group 50α and Group 50T + 50α; FC > 2 and *p < 0.05*; FC: Fold change.

In order to explore whether specific signaling pathways are regulated by TNF-α and TMAO in Hepa1-6 cells, we analyzed the transcriptomic alterations in the four treatment groups of Hepa1-6 cells after treatment for 24 h. The RNA sequencing data revealed 3741, 398, 3018 and 2674 differentially expressed genes (DEGs; FC > 2 and *p* < 0.05) in the untreated group (Group Ctrl), cells treated with 50 μM TMAO alone (Group 50T), cells treated with 50 ng/ml TNF-α alone (Group 50α), and cells treated with 50 ng/ml TNF-α and 50 μM TMAO in combination (Group 50T + 50α), respectively. Clustering identified 35 DEGs that were common to all four groups and the expression of 35 DEGs in four groups are shown in the heat map B (Figures 2A,B). In addition, to investigate genes involved in the ability of TMAO to amplify hepatocellular carcinoma cell proliferation and migration in the presence of TNF-α, we analyzed the expression differences in the 35 DEGs between Group 50α and Group 50T + 50α. The genes with the greatest differences were *POSTN*, *NAPB*, *LAYN*, *HTRA3*, *AANAT*, *ZFP850* and *AFM* (Figure 2C). Then, a volcano plot was generated to identify the most significantly differential genes in Group 50T + 50α compared with Group 50α (Figure 2D), which confirmed the previous heat map analysis.

### Associations Between the Identified DEGs and Survival Outcomes in Human Liver Cancer

**FIGURE 3:**
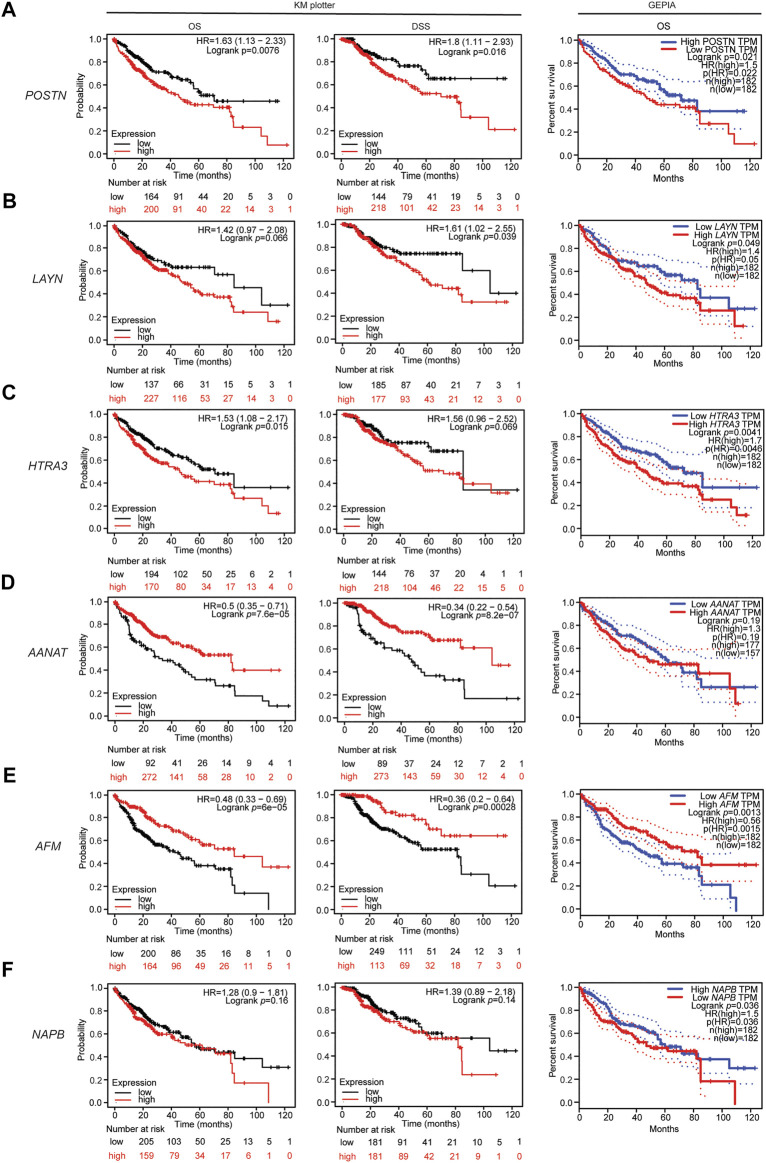
Associations between the DEGs and patient outcomes in liver cancer. Overall survival curve for patients with liver cancer in the GEPIA database (right) and overall survival and disease specific survival curves (left, center) for patients with liver cancer in the Kaplan-Meier plotter databases stratified by expression of **(A)** *POSTN*, **(B)** *LAYN*, **(C)** *HTRA3*, **(D)** *AANAT*, **(E)** *AFM*, and **(F)** *NAPB*.

Next, we investigated whether the expression of the identified DEGs correlates with the outcomes of patients with liver cancer using the Kaplan-Meier plotter database. Kaplan Meier plotter can be used to assess the effects of 54,000 genes (mRNA, miRNA, protein) on survival in several cancers based on microarray data; we focused on the overall survival (OS) and disease-specific survival (DSS) rates for liver cancer in this database. Interestingly, higher expression of *POSTN* (OS HR = 1.63, 95% CI = 1.13–2.33, *p* = 0.0076; DSS HR = 1.8, 95% CI = 1.11–2.93, *p* = 0.016; Figure 3A), *LAYN* (DSS HR = 1.61, 95% CI = 1.02–2.55, *p* = 0.039; Figure 3B) and *HTRA3* (OS HR = 1.53, 95% CI = 1.08–2.17, *p* = 0.015) were associated with poorer outcomes in liver cancer (Figure 3C). Moreover, lower expression of *AANAT* (OS HR = 0.5, 95% CI = 0.35–0.71, *p* = 7.6e-05; DSS HR = 0.34, 95% CI = 0.22–0.54, *p* = 8.2e-07; Figure 3D) and *AFM* (OS HR = 0.48, 95% CI = 0.33–0.69, *P* = 6e-05; DSS HR = 0.36, 95% CI = 0.2–0.64, *p* = 0.00028; Figure 3E) were strongly associated with poorer outcomes in liver cancer. In contrast, upregulation of *NAPB* was not associated with OS (HR = 1.28, 95% CI = 0.9–1.81, *p* = 0.16) or DSS (HR = 1.39, 95% CI = 0.89–2.18, *p* = 0.14) in liver cancer (Figure 3F). Moreover, upregulation of *LAYN* and upregulation of *HTRA3* were not significantly associated with OS (HR = 1.42, 95% CI = 0.97–2.08, *p* = 0.066) or DSS (HR = 1.56, 95% CI = 0.96–2.52, *p* = 0.069), respectively in liver cancer (Figures 3B,C). Overall, the survival analysis based on the DEGs suggests that the expression of *POSTN, HTRA3*, *LAYN, AFM* and *AANAT* are associated with the outcomes of patients with liver cancer.

In addition, the RNA sequencing data in the TCGA database were used to further analyze the associations of the identified DEGs with the outcomes of patients with liver cancer via GEPIA. High expression of *POSTN*, *LAYN*, *HTRA3* and *NAPB* were associated with poorer OS in liver hepatocellular carcinoma (LIHC; Figures 3A–C,F). Moreover, low *AANAT* expression and low *AFM* expression were associated with poorer OS in LIHC (Figures 3D,E). These results confirm that the *POSTN*, *NAPB*, *HTRA3*, *LAYN*, *AFM* and *AANAT* genes may contribute to tumorigenicity and lead to poorer patient outcomes in liver cancer.

### Associations Between the Identified DEGs and Neutrophil/Treg Infiltration in HCC

**FIGURE 4:**
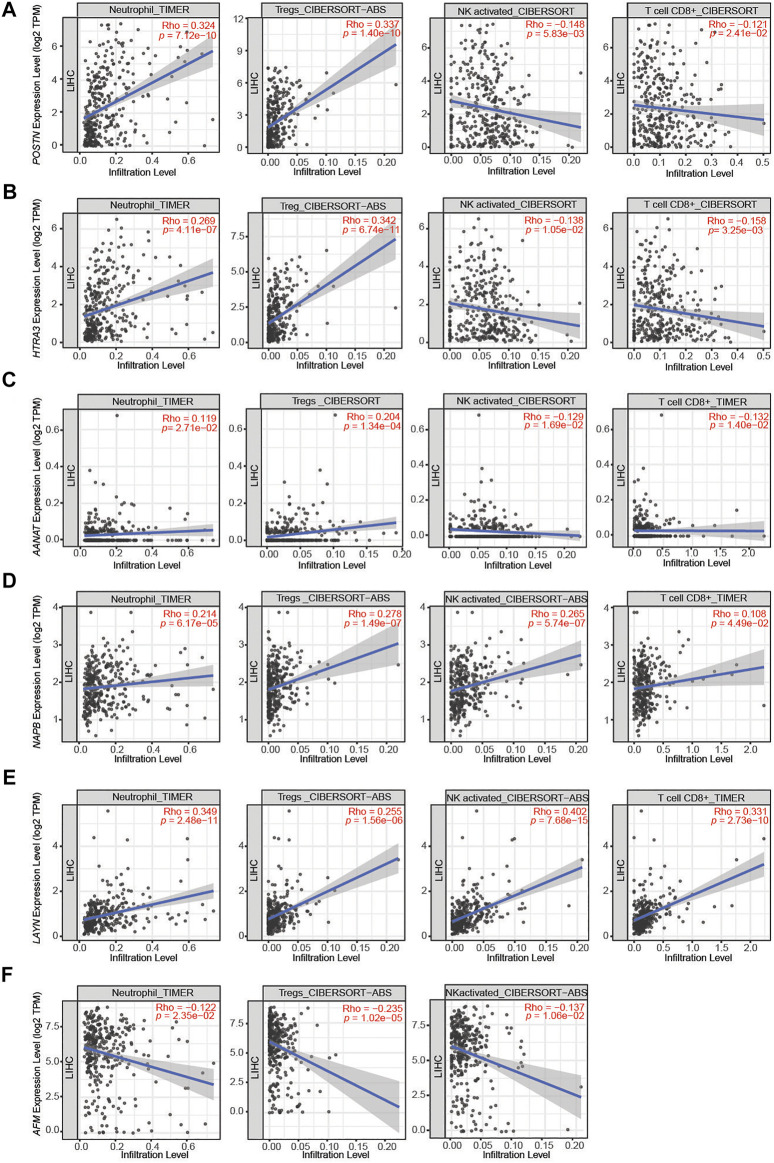
Correlations between the DEGs and immune cell infiltration in liver hepatocellular carcinoma (LIHC). **(A–C)** The expression levels of *POSTN*, *HTRA3* and *AANAT* significantly positively correlated with the levels of neutrophil and Treg infiltration and negatively correlated with the levels of activated NK cell and CD8^+^ T cell infiltration. **(D,E)** The expression levels of *NAPB* and *LAYN* significantly positively correlated with neutrophil, Treg, activated NK cell and CD8^+^ T cell infiltration. **(F)** The expression levels of *AFM* negatively correlated with the levels of neutrophil, Treg and activated NK cell infiltration.

To investigate the relationship between the DEGs identified in this study and the diverse variety of infiltrating immune cells in liver cancer, we explored the associations between the DEGs and immune marker sets for various immune cells, including neutrophils, regulatory T cells (Tregs), NK cells and CD8^+^ T cells, in LIHC using the TIMER database, using LIHC as the control. The immunosuppressed HCC tumor microenvironment was positively correlated with neutrophil and Treg infiltration and negatively correlated with activated NK cell and CD8^+^ T cell infiltration. After screening in the database, we found the expression levels of *POSTN*, *HTRA3* and *AANAT* significantly positively correlated with neutrophil and Treg infiltration and negatively correlated with activated NK cell and CD8^+^ T cell infiltration in LIHC (Figures 4A–C). Moreover, the expression levels of *NAPB* and *LAYN* significantly positively correlated with neutrophil, Treg, activated NK cell and CD8^+^ T cell infiltration (Figures 4D,E), whereas the expression of *AFM* negatively correlated with neutrophil, Treg and activated NK cell infiltration (Figure 4F). These results indicate that *POSTN* and *HTRA3* are positively associated with an inflammatory tumor microenvironment, which has been proven to promote tumor progression.

### TMAO Regulates the Expression of Prognosis-Related Genes in TNF-α Induced Inflammatory HCC

**FIGURE 5:**
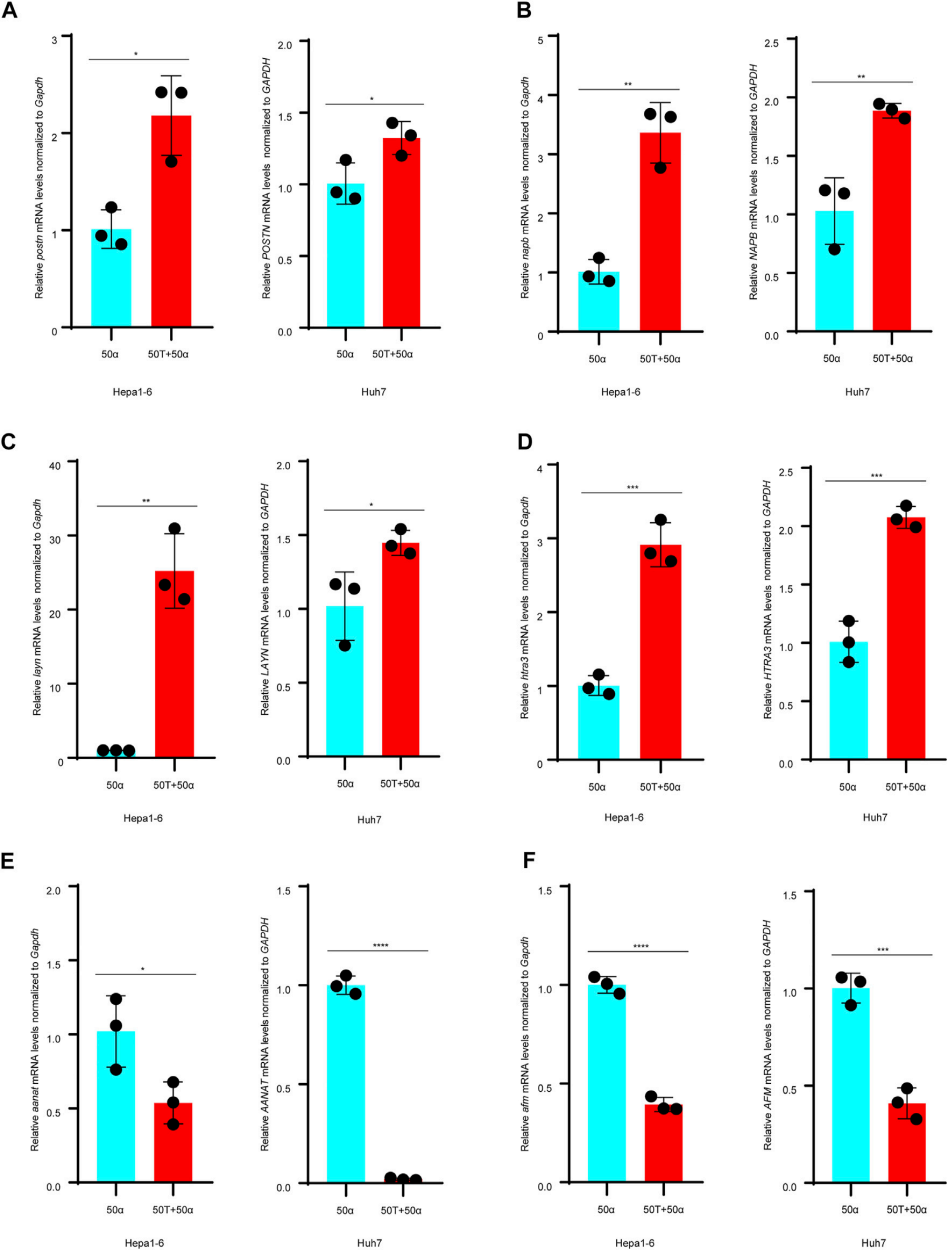
TMAO and TNF-α alter expression of the DEGs in Hepa1-6 cells and Huh7 cells. **(A–D)** Expression of the *POSTN*, *NAPB*, *LAYN* and *HTRA3* mRNAs were significantly upregulated in Hepa1-6 cells and Huh7 cells treated with 50 ng/ml TNF-α and 50 μM TMAO in combination compared to cells treated with 50 ng/ml TNF-α alone; results are mean ± SD (*n* = 3 per group); Student’s t-test. **(E,F)** On the contrary, 50 ng/ml TNF-α and 50 μM TMAO in combination significantly downregulated the expression of *AANAT* and *AFM* compared to cells treated with 50 ng/ml TNF-α alone; results are mean ± SD (*n* = 3 per group); Student’s t-test.

**FIGURE 6:**
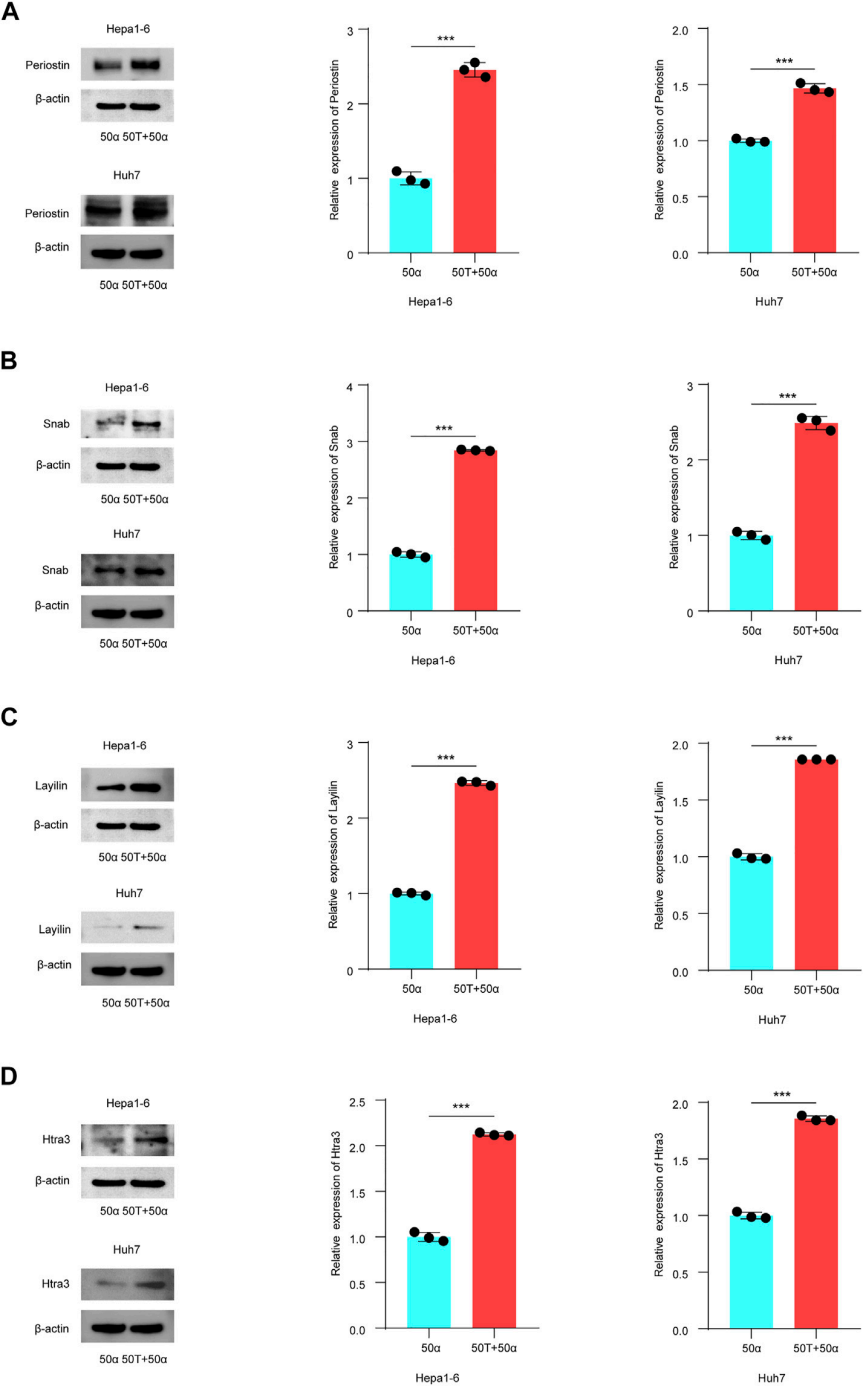
TMAO regulates the expression of DEGs related to patient outcome in TNF-α induced inflammatory HCC. **(A–D)** Western blotting was used to analyze the expression level of Periostin, Snab, Layilin and Htra3 in Hepa1-6 and Huh7 cells treated with 50 ng/ml TNF-α combined with 50 μM TMAO or 50 ng/ml TNF-α alone. Grayscale values were analyzed; data are mean ± SD (*n* = 3 per group); Student’s t-test; ***, *p* < 0.001.

To explore whether TMAO affects the expression of the DEGs identified to affect patient outcomes, Hepa1-6 and Huh7 cell were treated with 50 ng/ml TNF-α either alone or in combination with 50 μM TMAO. The expression of the genes and their products were quantified at the transcriptional and translational levels by RT–qPCR and western blotting. RT–qPCR suggested that TNF-α together with TMAO significantly upregulated the mRNA level of Periostin (*Postn*) compared to cells treated with TNF-α alone in Hepa1-6 cells; these changes were confirmed by western blotting (Figure 5A, 6A). Moreover, the mRNA level of Snab (*Napb*) was significantly increased by the combination of TNF-α and TMAO, with obvious upregulation at the transcriptional level (Figures 5B, 6B). TMAO in combination with TNF-α also significantly upregulated Layilin (*Layn*) and Htra3 (*Htra3*) at both the transcriptional and translational levels (Figures 5C,D, 6C,D). Moreover, afamin (*Afm*) and serotonin N-acetyltransferase (*Aanat*) were downregulated in both cell lines after treatment with TMAO and TNF-α (Figures 5E,F). Overall, the changes observed in the mouse cell lines mirrored the trends demonstrated in the transcriptomic analysis of human tumor samples in Figures 2C,D, and confirm that TMAO significantly upregulates Periostin, Snab, Layilin and Htra3 and downregulates afamin and serotonin N-acetyltransferase in liver cancer in the presence of TNF-α.

### TMAO Activates ILK/AKT/mTOR Signaling *via* Postn

*Postn*, which encodes Periostin, was the most significantly altered DEG in our transcriptomic profiling and the gene most significantly associated with the immunosuppressed tumor microenvironment in HCC. Previous studies confirmed that *Postn* is closely related to cancer progression and may promote tumor invasion through the ILK (Integrin-linked protein kinase)/RAC-alpha serine (AKT)/Serine/threonine-protein kinase mTOR (mTOR) pathway (Zhou et al., 2015; Chen et al., 2020; Jia et al., 2021). The ILK/AKT and mTOR signaling pathway regulates various aspects of cell development and is also implicated in the progression of human carcinomas (Ma et al., 2011; Jia et al., 2021; Pappa et al., 2021).

**FIGURE 7:**
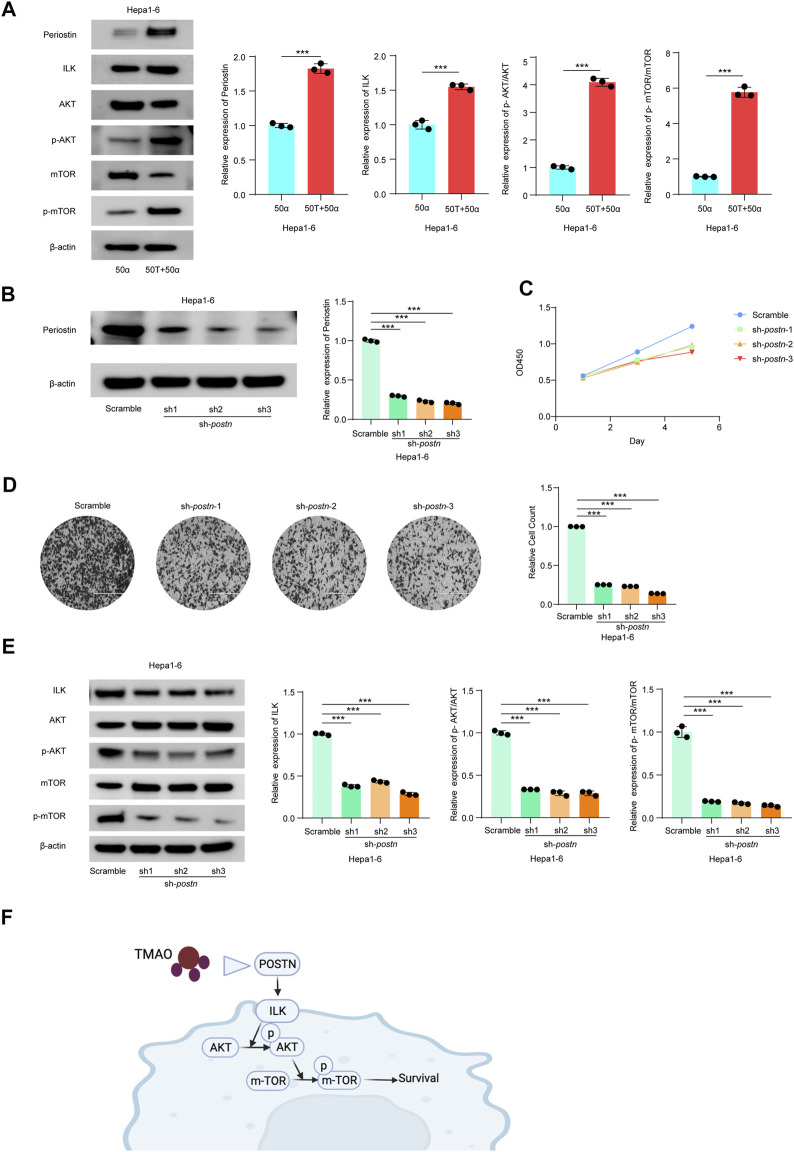
TMAO activates ILK/AKT/mTOR signaling via *Postn* in Hepa1-6 cells. **(A)** The translation levels of POSTN, ILK, AKT, p-AKT, mTOR and p-mTOR measured by western blotting in Hepa1-6 treated with 50 ng/ml TNF-α combined with 50 μM TMAO or 50 ng/ml TNF-α alone. Grayscale values were analyzed; data are mean ± SD (*n* = 3 per group); Student’s t-test; ***, *p* < 0.001. **(B)** POSTN translation levels in Hepa1-6 cells infected with pseudovirus expressing sh-*postn* (sh1, sh2, sh3) plasmids were compared with the Hepa1-6 cells infected with pseudovirus expressing scramble plasmids measured by western blotting. Grayscale values were analyzed; data are mean ± SD (*n* = 3 per group); one-way ANOVA with Bonferroni’s test; ***, *p* < 0.001. **(C)** CCK-8 assay of the effect of knocking down *Postn* on the proliferation of Hepa1-6 cells; data are mean ± SD (*n* = 3 per group); one-way ANOVA with Bonferroni’s test; ***, *p* < 0.001. **(D)** Transwell assays of the effect of knocking down *Postn* on the invasive abilities of Hepa1-6 cells. Scale bars = 100 μm; Data were analyzed by ImageJ; data are mean ± SD (*n* = 3 per group); one-way ANOVA with Bonferroni’s test; ***, *p* < 0.001. **(E)** Western blots of the effects of knocking down *Postn* on the protein levels of Postn, ILK, AKT, p-AKT, mTOR and p-mTOR in Hepa1-6 cells. Grayscale values were analyzed; data are mean ± SD (*n* = 3 per group); one-way ANOVA with Bonferroni’s test; ***, *p* < 0.001. **(F)** Schematic diagram of the role of TMAO in activation of the ILK/AKT/mTOR signaling pathway via POSTN.

**FIGURE 8:**
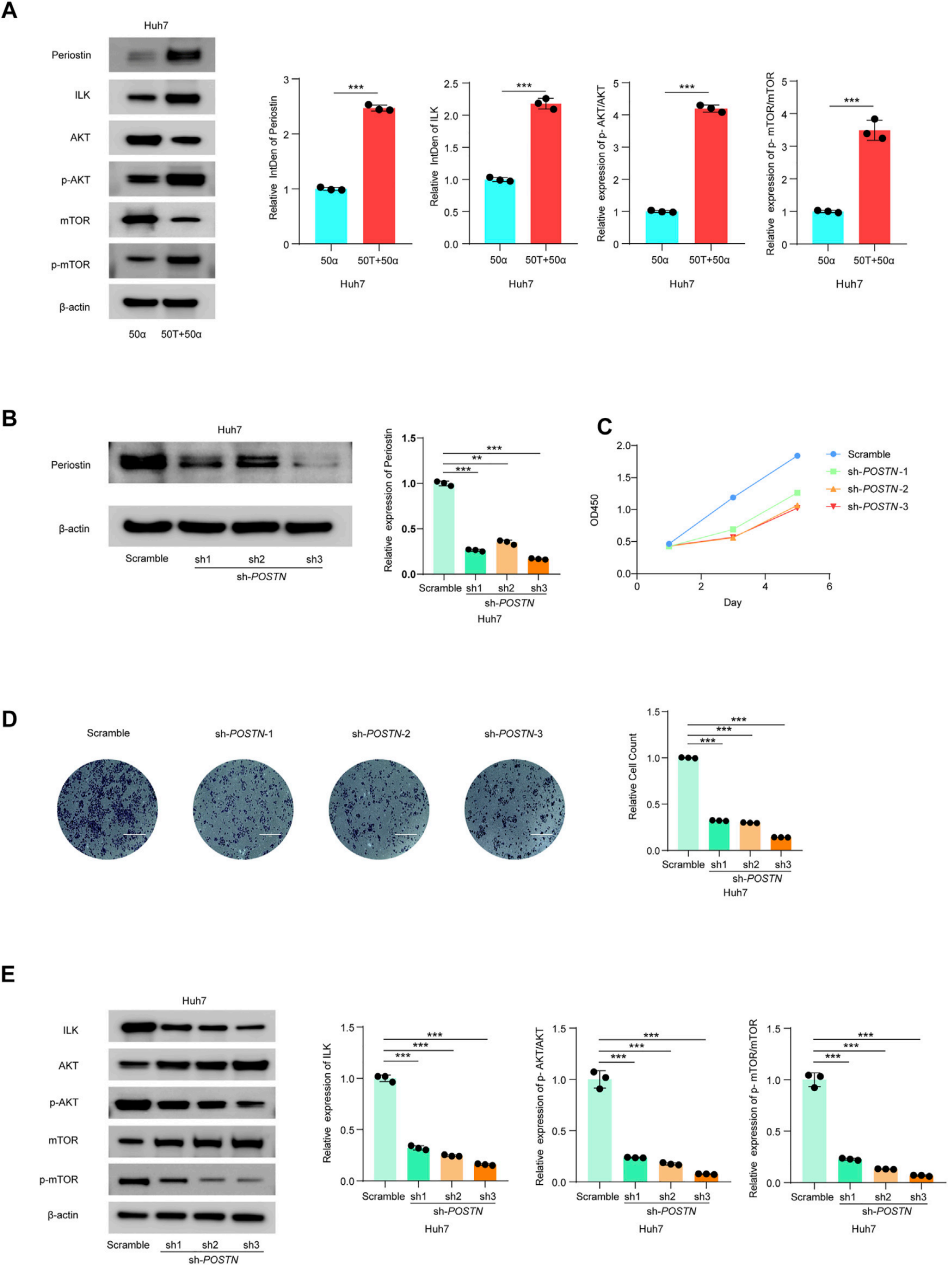
TMAO activates ILK/AKT/mTOR signaling via *Postn* in Huh7 cells. **(A)** The translation levels of POSTN, ILK, AKT, p-AKT, mTOR and p-mTOR measured by western blotting in Huh7 cells treated with 50 ng/ml TNF-α combined with 50 μM TMAO or 50 ng/ml TNF-α alone. Grayscale values were analyzed; data are mean ± SD (*n* = 3 per group); Student’s t-test; ***, *p* < 0.001. **(B)** *POSTN* translation levels in Huh7 cells infected with pseudovirus expressing sh-*POSTN* (sh1, sh2, sh3) plasmids were compared with the Huh7 cells infected with pseudovirus expressing scramble plasmids measured by western blotting. Grayscale values were analyzed; data are mean ± SD (*n* = 3 per group); one-way ANOVA with Bonferroni’s test; ***, *p* < 0.001. **(C**) CCK-8 assay of the effect of knocking down *POSTN* on the proliferation of Huh7 cells; data are mean ± SD (*n* = 3 per group); one-way ANOVA with Bonferroni’s test; ***, *p* < 0.001. **(D)** Transwell assays of the effect of knocking down *POSTN* on the invasive abilities of Huh7 cells. Scale bars = 100 μm; Data were analyzed by ImageJ; data are mean ± SD (*n* = 3 per group); one-way ANOVA with Bonferroni’s test; ***, *p* < 0.001. **(E)** Western blots of the effects of knocking down *POSTN* on the protein levels of Postn, ILK, AKT, p-AKT, mTOR and p-mTOR in Huh7 cells. Grayscale values were analyzed; data are mean ± SD (*n* = 3 per group); one-way ANOVA with Bonferroni’s test; ***, *p* < 0.001.

Thus, we investigated the effects of TMAO and TNF-α on activation of the ILK/AKT and mTOR signaling pathway *in vitro.* Exposure to 50 ng/ml TNF-α combined with 50 μM TMAO significantly increased the levels of ILK, phosphorylated AKT and mTOR phosphorylation, and also increased expression of Postn compared to cells treated with 50 ng/ml TNF-α alone. Conversely, TMAO and TNF-α reduced the levels of AKT, and significantly decreased mTOR expression (Figures 7A, 8A).

To further study the potential oncogenic activity of Periostin in Hepa1-6 cells, knockdown studies were performed using three short hairpin RNAs (shRNAs) with different sequences targeting *Postn*. Transfection of sh-*postn* (sh1, sh2, sh3) into Hepa1-6 cells and Huh7 cells effectively reduced the expression of *Postn* (Figures 7B, 8B). The CCK-8 cell proliferation assay showed that knockdown of *Postn* significantly reduced the proliferation of Hepa1-6 cells at 72 h compared to cells transfected with the scrambled control shRNA (Figure 7C). Moreover, Transwell assays demonstrated that the migratory capacity of Hepa1-6 cells was significantly reduced by knockdown of *Postn* (Figure 7D). The same situation also exists in the Huh7 cell line (Figures 8C,D). Collectively, these results suggest that *Postn* promotes the proliferation and migration of Hepa1-6 cells and Huh7 cells.

In addition, knockdown of *Postn* downregulated ILK, phosphorylated AKT and mTOR phosphorylation in Hepa1-6 cells and Huh7 cells, and upregulated AKT and mTOR expression (Figures 7E, 8E). Overall, these results indicate that TMAO promotes cell proliferation and migration by activating the ILK/AKT/mTOR pathway via Periostin in Hepa1-6 cells and Huh7 cells (Figure 7F).

## Discussion

The cometabolite TMAO plays an important role in inflammatory diseases. However, the relationship between TMAO and HCC has received little study. Here, we report that TMAO plays an important role in promoting TNF-α-induced inflammatory liver cancer by enhancing hepatocyte proliferation, migration and invasion. Moreover, TMAO upregulates *POSTN*, *NAPB*, *LAYN*, and *HTRA3* in liver cancer, and high expression levels of these genes are closely associated with suppression of the immune microenvironment and patient survival outcomes in liver cancer. Furthermore, we found that TMAO upregulates *POSTN* and activates the ILK/AKT/mTOR pathway, which induces tumor proliferation and migration. These results provide the first evidence of the mechanism of action of TMAO in TNF-α-induced inflammatory liver cancer.

Periostin (encoded by *POSTN*) is a matricellular protein that functions as critical factor in the development of hepatic inflammation, fibrosis, liver cirrhosis and liver cancer (Kudo, 2011; Huang et al., 2015; Chen et al., 2020). Upregulation of POSTN promotes cell migration and invasion and is also associated with the epithelial-mesenchymal transition (EMT) in clinical HCC tissues (Chen et al., 2019). We found that the proliferation and migratory capacity of Hepa1-6 cells were significantly decreased after knockdown of *Postn*. In addition, downregulation of *POSTN* reduced the tumor-forming ability of HCC cell lines in xenograft mouse models (Chen et al., 2021). Moreover, *POSTN* mRNA expression positively correlated in tumor tissues, but not in non-tumor tissues (Kongkavitoon et al., 2018). We also confirmed that *POSTN* was the most significantly altered differential gene in the inflammatory hepatocellular carcinoma environment (Figure 2C). This evidence strongly suggests POSTN is involved in the complex mechanisms that lead to liver cancer.

*LAYN* is a key gene that regulates T cell function. Overexpression of *LAYN* in human blood CD8^+^ T cells significantly decreased IFN-γ production, which indicates that *LAYN* represses CD8^+^ T cell function (Zheng et al., 2017). Additionally, *LAYN* is associated with the levels of immune infiltration (CD8^+^ T cells, CD4^+^ T cells, macrophages and neutrophils) as well as patient prognosis in various cancers, especially colon cancer and gastric cancer (Pan et al., 2019). We found a significant positive correlation between neutrophils and Tregs infiltration and *LAYN* expression level. The high expression of *LAYN* was associated with poor prognosis in HCC patients. This suggests that *LAYN* is fully involved in the immune infiltration of liver cancer and plays an important role in the development of tumor. A study of the extracellular matrix in the HCC microenvironment suggested that *HTRA3* and four other genes were significantly associated with immune cell infiltration and could be used as genetic markers for a prognostic score (Liu et al., 2020). Similarly, we demonstrated a positive association between HTRA3 and the inflammatory tumor microenvironment. Taken together, these two genes have great potential as new therapeutic targets for liver cancer.

Traditional surgical treatments or drug therapies for HCC have strict indications and limited therapeutic effects. Therefore, a new therapeutic strategy is needed to improve the prognosis of patients with HCC. With the discovery of immune checkpoint mechanisms, the application of immunotherapy in cancer is gradually being explored, and liver cancer has a higher immunotherapeutic potential as an inflammatory-driven disease (Okusaka and Ikeda, 2018). Tumor cell proliferation, invasion and metastasis are regulated by many factors. The interaction of multiple immune cells in complex pathways leads to the occurrence of tumors. We found that DEGs with significant upregulation of TMAO were positively correlated with upregulation of neutrophils and Tregs in the inflammatory hepatocellular carcinoma environment. Neutrophils represent the majority of inflammatory cells in solid tumors (Shen et al., 2014). Tumor-associated neutrophils participate in the tumor microenvironment (TME) by producing cytokines and chemokines that influence the growth of tumor cells. Autophagy is enhanced by activation of ERK1/2, P38, and NF-κB signaling after neutrophil infiltration. This strongly increases neutrophil survival and exerts tumor-promoting effects in HCC (Li et al., 2015; Masucci et al., 2019). Additionally, neutrophil-conditioned medium was shown to increase the migration of HCC cells or mouse macrophages and Tregs. It showed that neutrophils recruit macrophages and Tregs into HCC to promote tumor growth and progression (Zhou et al., 2016). Tregs are recruited into the TME and enable tumor cells to evade immune surveillance, and excessive Treg activity can lead to cancer by inhibiting the anti-tumor immune response (Lee, 2017; Tanaka and Sakaguchi, 2019). Therefore, neutrophils and Tregs promote tumorigenesis through complex mechanisms. TMAO may promote the development of inflammatory liver cancer through these pathways. Therapeutic regulation of Treg function has become a promising treatment approach (Wang et al., 2013). Immunotherapy targeting the TMAO pathway is an effective clinical treatment for HCC patients without surgical indications.

ILK regulates cell growth, motility and differentiation, and plays biological functions through a variety of signaling pathways (Hannigan et al., 1996; Riaz et al., 2013; Jackson et al., 2015; Shen et al., 2016; Piedra-Quintero et al., 2018; Huang et al., 2020). ILK is widely overexpressed in different cancers and its upregulation is closely related to tumor grade and survival (Zheng et al., 2019). Moreover, the ability of ILK to promote tumor proliferation, invasion and metastasis has been widely studied (Oloumi et al., 2010; Taylor et al., 2011). The present study demonstrated that TMAO significantly increased ILK levels, phosphorylated AKT and phosphorylation of mTOR. *POSTN* expression was positively correlated with ILK pathway activation. These results suggest that ILK and related pathways play an important role in the pathophysiological process of liver cancer. ILK is closely related to tumor grade and survival and its up-regulation is associated with poor prognosis. Therefore, it is an important indicator in cancer diagnosis and prognosis. In conclusion, periostin activates the proliferation and migration of tumor cells by the ILK/AKT/mTOR pathway. TMAO promotes the occurrence of inflammatory hepatocellular carcinoma by upregulating *POSTN*.

Studies have shown a direct mechanistic link between the metabolism of dietary nutrients such as choline and the production of TMAO. The precursors of TMAO include carnitine, lecithin, choline and betaine, which are abundant in red meat, liver, fish, milk, wheat bran and spinach (Hamlin et al., 2013). Dairy intake positively correlates with the plasma TMAO concentration (Rohrmann et al., 2016). The levels of TMAO can be easily and effectively reduced by controlling the types and amounts of food consumed, which may provide an effective, low-cost method among patients with liver cancer. However, microorganisms in the gut must first convert nutrients into TMA before TMAO can be produced (Koeth et al., 2013), thus reducing the TMA-targeting pathway may also lower TMAO levels. One study identified a structural analogue of choline, 3,3-dimethyl-1-butanol (DMB), inhibited formation of TMA by cultured microorganisms and in various microbial lyases, and reduced the level of TMAO in mice fed a high choline or carnitine diet (Wang et al., 2015; Chen K. et al., 2017). DMB has been widely studied and shown to reverse or prevent various heart and brain disorders and problems in other tissues (Yang W. et al., 2019; Mao et al., 2021). However, the potential of DMB as a treatment for liver cancer needs to be further explored. Furthermore, the important role of *POSTN* in HCC makes it a potential therapeutic target and targeted therapy for *POSTN* will provide new ideas for immunotherapy for HCC.

## Data Availability

The data presented in the study are deposited in the ‘GEO database’ repository, accession number GSE185586.
