# Supplementary material for: Integrated Analysis Reveals the Gut Microbial Metabolite TMAO Promotes Inflammatory Hepatocellular Carcinoma by Upregulating POSTN
Source: Front Cell Dev Biol. 2022 May 23;10:840171. doi: 10.3389/fcell.2022.840171 (PMC9167932; doi:10.3389/fcell.2022.840171)
Supplement: Supplementary file 1 [file Table1.pdf]

Table S1

Gene		Primer sequences
Postn	Forward	5' GCCTTAGCGACCTCTACAAT 3'
	Reverse	5' TAGCCGTCCGATACACAA 3'
Napb	Forward	5' GTTACTGGGGCTAGAGATGCC 3'
	Reverse	5' GGCCGTGAACAAAGCAGTAA 3'
Layn	Forward	5' GTCAGGTCGACCACTTTCCC 3'
	Reverse	5' TGAATTGGCTTTTGCAGGGTG 3'
Htra3	Forward	5' GCCAAGACTGGTCCCATGAA 3'
	Reverse	5' GCCAAGGCTACCAATGGTCT 3'
Aanat	Forward	5' GCGCCACACACTCCCTGCCA 3'
	Reverse	5' GCCCTTGCCCTGCTGCCGGAAG 3'
Afm	Forward	5' CTGGCCATCATCGCGTTTTC 3'
	Reverse	5' AAGGGGTTCCTTCTGGCAAC 3'
POSTN	Forward	5' GCGAGATCATCAAGCCAGCAGAG 3'
	Reverse	5' ATGTCCAGTCTCCAGGTTGTGTCA 3'
NAPB	Forward	5' GGAAACGCATTTTGTCAGGCA 3'
	Reverse	5' TTGCCCCAACCTGCTCATAG 3'
LAYN	Forward	5' ACAGAGCTGACAACACCTGTA 3'
	Reverse	5' GATGTAGGCCAGATTCAAGGC 3'
HTRA3	Forward	5' CGTGGTGTCCAGCAACAGTGC 3'
	Reverse	5' CCGTGTGATGCGGTCTGAGGG 3'
AANAT	Forward	5' CAAAATCAGAAGGGAACAGTA 3'
	Reverse	5' TGGATGACAAATAGACAAGA 3'

AFM	Forward	5' GCTCACGAAGATAGCTCCCC 3'
	Reverse	5' AGCCTTCCCTTTGGTGCTTT 3'

All primer sequences are derived from primer pick(https://www.ncbi.nlm.nih.gov/tools/primer-blast/).
